# Initial Results from the Royal College of Radiologists' UK National Audit of Anal Cancer Radiotherapy 2015

**DOI:** 10.1016/j.clon.2016.10.005

**Published:** 2017-03

**Authors:** R. Muirhead, K. Drinkwater, S.M. O'Cathail, R. Adams, R. Glynne-Jones, M. Harrison, M.A. Hawkins, D. Sebag-Montefiore, D.C. Gilbert

**Affiliations:** ∗ CRUK MRC Oxford Institute for Radiation Oncology, University of Oxford, Oxford, UK; † The Royal College of Radiologists, London, UK; ‡ Oxford University Hospitals NHS Trust, Department of Oncology, Churchill Hospital, Oxford, UK; § Cardiff University Department of Cancer and Genetics and Velindre Hospital, Cardiff, UK; ¶ Mount Vernon Centre for Cancer Treatment, Mount Vernon Hospital, Northwood, UK; || University of Leeds, Cancer Research UK Leeds Centre, St. James's University Hospital, Leeds, UK; ∗∗ Sussex Cancer Centre, Royal Sussex County Hospital, Brighton, UK

**Keywords:** Acute toxicity, anal cancer, audit, IMRT

## Abstract

**Aims:**

UK guidance was recently developed for the treatment of anal cancer using intensity-modulated radiotherapy (IMRT). We audited the current use of radiotherapy in UK cancer centres for the treatment of anal cancer against such guidance. We describe the acute toxicity of IMRT in comparison with patient population in the audit treated with two-phase conformal radiotherapy and the previous published data from two-phase conformal radiotherapy, in the UK ACT2 trial.

**Materials and methods:**

A Royal College of Radiologists' prospective national audit of patients treated with radiotherapy in UK cancer centres was carried out over a 6 month period between February and July 2015.

**Results:**

Two hundred and forty-two cases were received from 40/56 cancer centres (71%). In total, 231 (95%) underwent full dose radiotherapy with prophylactic nodal irradiation. Of these, 180 (78%) received IMRT or equivalent, 52 (22%) two-phase conformal (ACT2) technique. The number of interruptions in radiotherapy treatment in the ACT2 trial was 15%. Interruptions were noted in 7% (95% confidence interval 0–14%) of courses receiving two-phase conformal and 4% (95% confidence interval 1–7%) of those receiving IMRT. The percentage of patients completing the planned radiotherapy dose, irrelevant of gaps, was 90% (95% confidence interval 82–98%) and 96% (95% confidence interval 93–99%), in two-phase conformal and IMRT respectively. The toxicity reported in the ACT2 trial, in patients receiving two-phase conformal in the audit and in patients receiving IMRT in the audit was: any toxic effect 71%, 54%, 48%, non-haematological 62%, 49%, 40% and haematological 26%, 13%, 18%, respectively.

**Conclusions:**

IMRT implementation for anal cancer is well underway in the UK with most patients receiving IMRT delivery, although its usage is not yet universal. This audit confirms that IMRT results in reduced acute toxicity and minimised treatment interruptions in comparison with previous two-phase conformal techniques.

## Highlights

• Most UK centres deliver IMRT to all or selected anal cancer patients.
• IMRT reduced any grade 3 or 4 acute toxic effect in comparison with two-phase conformal from 54% to 48%.
• IMRT reduced interruptions in treatment in comparison with two-phase conformal from 7% to 4%.
• Completion rate of planned radiotherapy dose in comparison with two-phase conformal from 90% to 96%.

## Introduction

Anal cancer is a relatively rare tumour with an increasing incidence [1]. It is associated with infection with high-risk subtypes of human papilloma viruses [2]. The ACT2 study set the standard for radical chemoradiotherapy (CRT) in anal squamous cell carcinoma in the UK, with a 3 year disease-free survival of 73% [3]. However, the radiotherapy techniques available at the time of trial design, large anterior–posterior/posterior–anterior fields, were associated with significant acute toxicity, particularly in the skin and perineum. Although relatively modest total radiation doses were used, this toxicity often entailed long breaks in treatment associated with worse oncological outcomes and considerable late pelvic radiation morbidity [4], [5], [6], [7], [8].

Intensity-modulated radiotherapy (IMRT) conforms doses around irregular volumes using multiple beams and varying dose rates. This minimises dose to normal organs with the aim of reducing toxicity. The Radiation Therapy Oncology Group (RTOG) 0529 single arm phase II study confirmed reduced acute toxicity with IMRT in radical anal CRT; when retrospectively compared with the previous RTOG 9811 trial, where radiotherapy was delivered conformally using a shrinking field technique [9]. RTOG 0529 delivered 54 Gy and 45 Gy in 30 fractions to primary tumour and prophylactic lymph nodes, respectively, in locally advanced disease and 50 Gy and 42 Gy in 28 fractions, respectively, to early disease.

In 2012, the UK Department of Health recommended that all patients who could benefit from reduced treatment toxicity through the use of IMRT should be offered this treatment [10]. However, the implementation of IMRT for any given indication brings a number of challenges. Converting ACT2 radiotherapy to an IMRT protocol required consideration of doses, volumes and technique [11]. Implementation without due care and quality control can result in geographical miss; potentially reducing cure rates and/or increasing toxicity. Some authors have questioned the uncritical adoption of IMRT, raising concerns regarding the steep and long learning curve required for the technique to be perfected and the lack of quality assurance [12]. Delhorme *et al.* [13] reported improved outcomes with the use of guidelines. However, in RTOG 0529, despite the stringent protocolised setting of a clinical trial, 81% of plans submitted for central review were rejected at first review and 46% required multiple revisions [9]. They also reported correlations between minor or major deviations from protocol and outcomes; patients who had a minor or major deviation in dose to small bowel had a increased rate of Grade 2+ toxicity. As such, it is vital in the multicentre implementation of IMRT to agree detailed homogeneous delivery guidelines, encourage education and mentoring; and incorporate adequate quality assurance.

To investigate the implementation of IMRT in anal cancer in the UK, the Royal College of Radiologists (RCR) surveyed 58 centres in November 2013, requesting information on current radiotherapy delivery techniques used for anal cancer and the ability and time frames for implementing IMRT [14]. The results showed that IMRT implementation had begun in a sporadic manner with different delineation, doses and constraints being used. The results of the survey are available as supplementary material. This highlighted the difficulties of implementing a new technique in a rare cancer with limited supporting evidence and few treating clinicians. As such, a working group of specialist clinicians in anal cancer was convened, supported by the Anorectal Clinical Studies Sub Group (CSG) of the National Cancer Research Institute (NCRI), to develop consensus guidance detailing standard radiotherapy volume delineation, dose and fractionation based on the volumes and doses used in the ACT2 study [15]. This was presented at the annual NCRI and other meetings [16] and highlighted in an editorial [14]. A national audit was initiated in order to assess the implementation of this challenging technique. Furthermore, future clinical trials will require an IMRT platform, and as such an IMRT solution was required, ideally with implementation and an audit of implementation before the development of further studies.

The audit presented here was carried out 2 years after the initial survey with the aims:

(i) To benchmark the national delivery of radiotherapy in anal cancer and identify potential for improvements.
(ii) To compare UK practice with National Comprehensive Cancer Network (NCCN) and ESMO-ESSO-ESTRO guidance.
(iii) To assess whether the number of patients receiving IMRT are in keeping with National Radiotherapy Implementation Group (NRIG) IMRT recommendations.
(iv) To document the compliance with suggested UK IMRT guidance.
(v) To describe the acute toxicity of IMRT as per UK guidance in comparison with previous ACT2 published toxicity.
(vi) To provide a UK-wide standard of care to optimise the opportunities for clinical research and improvements in this disease in the future at a national level.

## Materials and Methods

We aimed to collect prospective data on all patients with a diagnosis of anal cancer, in all UK National Health Service cancer centres, starting radiotherapy over a 6 month period from 9 February to 27 July 2015. Patient demographic data included the age and gender of patient, whether or not they underwent a pre-treatment stoma, HIV and smoking status. Tumour demographic data included pathology, level of differentiation, stage and site of primary and lymph nodes. Details of chemotherapy and radiotherapy treatment and weekly CTC acute toxicity (v4.03, 2010) during treatment were collected. RTOG grading was used for skin toxicity. Finally patients were asked to complete a European Organization for Research and Treatment of Cancer (EORTC) questionnaire to document the baseline patient-reported outcomes of disease and treatment symptoms [17], [18]. Data points collected are documented within supplementary material. A specifically developed web-based data collection form was constructed using Snap WebHost Professional survey software. The data form was reviewed by the RCR Clinical Oncology Audit Committee and, after revision, piloted in five centres. Clinical oncology audit leads acted as points of contact between the RCR and participating centres.

Data were reviewed by RM and DG. On review of the submitted data, grade 3 toxicity was noted if: it resulted in an admission, an interruption or discontinuation of chemotherapy or radiotherapy, or was noted as grade 3 in the weekly grading. Where a total white cell count had been submitted instead of a neutrophil count, the white cell count was divided by two to calculate an approximate neutrophil count that was used for analysis (as neutrophils make up approximately 50% of the white cell count, this method was deemed an appropriate estimate of the neutrophil count [19]). In the case of discordance between lymph node location and stage, the stage was manually corrected in keeping with the site and number of lymph nodes in accordance with AJCC 7th edition, 2010 [20]. The maximum toxic effect grade was used for each patient and each event type. An interruption in radiotherapy treatment was defined as any extension to the treatment, >2 days over the planned overall treatment time, as per RCR guidance on interruptions suggesting that any gap over 2 days requires compensation [21].

Data analysis was undertaken using Microsoft Office Excel 2013 (Microsoft Corporation, CA, USA) and Graphpad (www.graphpad.com). All analyses are intention-to-treat unless otherwise specified.

## Results

Two hundred and forty-two cases were received from 40 of 56 (71%) UK radiotherapy centres. Two centres replied that they refer anal cancer patients elsewhere. The number of cases received from contributing centres varied from one to 13 (median six). Cases were categorised as either ACT2 style (for the purposes of this audit termed ‘two-phase conformal CRT’[AQ1]) or IMRT. Within the two-phase conformal CRT group and the IMRT groups, the number of cases varied from one to 11 (median three) and one and 12 (median three), respectively. This is because eight centres were contributing both two-phase conformal CRT and IMRT data. Of the four centres submitting ≥10 cases; three centres used solely IMRT for radical treatment, one used solely two-phase conformal CRT. Table 1, Table 2 detail patient and tumour demographics, respectively. The groups were relatively well balanced with non-significant differences in gender (11 men versus 41 women in the two-phase conformal CRT group and 47 men versus 110 women in the IMRT treated group; *x*^2^ *P* = 0.284), pre-treatment stoma formation (11/52 = 21% with two-phase conformal CRT and 24/157 = 15% in the IMRT group, *x*^2^ *P* = 0.391) and equivalent T and N stages. There was a significant difference in the proportion that were confirmed smokers (21/52 = 40% in the two-phase conformal CRT and 38/157 = 24% in the IMRT group; *x*^2^ *P* = 0.033). Although exact proportions differed, rates of T3/4 were similar in both groups (48% versus 47%). Slightly more patients treated with two-phase conformal CRT were known M1 (8% versus 2%) or unrecorded M stage (10% versus 4%).

**Fig 1:**
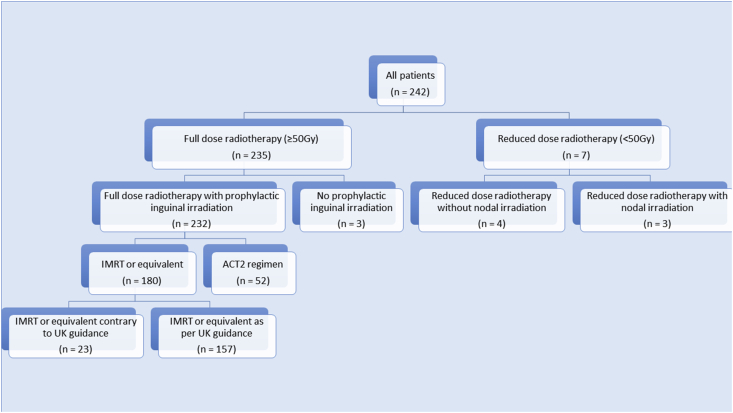
Flow diagram of radiotherapy doses, volumes and techniques.

In total, 232 (96%) patients received full dose radiotherapy to primary and prophylactic nodes. Of the 40 centres, 38 treated at least one patient with IMRT. Of the seven patients who had radiation to a reduced dose, one received treatment with palliative intent, three had T1 tumours or T2 tumours after excision, one was frail and elderly, two were elderly with a T1 or T2 post-excision tumour. Seven of the 242 (3%) did not undergo prophylactic inguinal node irradiation; four were T1, two after excision of a T2 and one patient chose to discontinue treatment early due to toxicity. Of those undergoing full dose inverse planned radiotherapy to primary tumour and all prophylactic nodes, 157 (87%) underwent treatment as described in the UK guidance document. Of those who did not receive treatment as per guidance; 21 (12%) received doses based on the RTOG 0529 publication, one delivered 50 Gy in 25 fractions and the last delivered a two-phase technique with ACT2 doses to the nodes in 17 fractions. Figure 1 illustrates the breakdown of radiotherapy doses, volumes and techniques.

Chemotherapy regimens are given in Table 3. Those patients who did not receive chemotherapy were due to frailty (one), T1 disease (one), age (one), the palliative nature of radiotherapy (one), ongoing pelvic abscesses (one), cardiac history and advancing age (one). In two patients (aged 70 and 81 years), no reason for withholding chemotherapy was given.

For the purposes of assessment and comparisons of acute toxicity, four groups were assessed: (A) data from the ACT2 publication; (B) all patients in the audit; (C) patients in the audit treated with full dose radiotherapy including inguinal nodes, using two-phase conformal CRT; and (D) patients in the audit, treated with full dose radiotherapy including inguinal nodes, with IMRT using UK guidance. The data on gaps and discontinuation were based on the whole dataset. The acute toxicity assessment was based on 199 and 192 patients where complete data were submitted for non-haematological and haematological toxicity, respectively. Table 4 details the toxicity.

The median treatment time in the ACT2 trial was 38 days and in the three groups in the audit was 39 days (all patients), with 39 and 38 days for two-phase conformal CRT and IMRT, respectively. The number of interruptions in radiotherapy treatment was 15% in ACT2 and in the audit groups 7%, 7% and 4%. The number of patients completing the planned radiotherapy dose (irrelevant of gaps) in the audit groups were 90% and 96%. The number of patients completing chemotherapy was 77% in ACT2 and in the audit groups 83%, 88%, 83%, respectively. Although these results were an improvement over those reported in ACT2 in terms of reduced interruptions and improved completion of treatment, differences between patients treated with two-phase conformal CRT and IMRT were not statistically different (Fishers exact test *P* = 0.3021 for interruptions, *P* = 0.1777 for completion of radiotherapy and *P* = 0.667 for completion of chemotherapy). Treatment-related deaths were <1% in ACT2 and <1%, 4%, and 0% in the audit groups. These are documented in Table 5.

## Discussion

To our knowledge no prospective, comprehensive audit of anal cancer practice has occurred to date. This audit gives a comprehensive representation of current anal radiotherapy practice, over a short time period, across the UK. Although the ACT studies captured valuable patient and tumour demographic data in a similar population, the data captured in a clinical trial tends to reflect a fitter population with a higher performance status, which may not represent routine clinical practice.

A number of factors prompted a national audit. The RCR survey showed the sporadic and heterogeneous implementation of IMRT in independent centres without any uniformed controlled roll-out, raising concerns due to the quality assurance problems encountered in the RTOG 0529 trial. The implementation of IMRT around the UK was carried out with informal mentoring between centres rather than a formal quality assurance programme. The planned collection of outcome data for audit patients will report IMRT outcomes. This acts as an objective quality assurance tool as if the delivery was carried out appropriately the outcomes should be comparable with two-phase conformal [AQ1] treatment. Furthermore, the forthcoming PLATO trial (PersonaLising Anal cancer RadioTherapy dOse, ISRCTN88455282) [22], [23], funded by Cancer Research UK, will require IMRT for anal cancer as standard. This is a platform of studies incorporating ACT3 (testing low dose involved field CRT for patients with close surgical margins); ACT4, a randomised phase II trial comparing standard CRT with reduced dose CRT in patients with T1–2 (up to 4 cm) N0 disease and ACT5, a seamless pilot/phase II/III trial comparing standard CRT dose escalation using a synchronous integrated boost.

The patient demographics are similar to those documented in ACT2 with the exception of an older population in the audit as expected due to the inclusion of all patients irrespective of their performance status, co-morbidity and treatment intent. Seventeen per cent of patients had an unknown smoking status. In anal cancer, smoking status is a prognostic factor [24], [25] and the toxicity of radiotherapy treatment is reported to be higher in those who continue to smoke in other tumour types [26], [27], [28], [29]. Therefore it is vital that smoking status is documented and smoking cessation advice given. The smoking rate in the audit (24%) exceeds the national smoking incidence of 19% [30]. The imbalance between the two-phase conformal CRT group (21/52 confirmed current smokers as opposed to only 38/119 in the group receiving IMRT) will be important when analysing outcome data. The next step is further investigation into the influence of smoking and work to assess and improve, if appropriate, counselling regarding smoking cessation. Another possible prognostic factor is the histological differentiation of the tumour [31], [32], [33]. In 20% of cases this was not reported or available and another factor that could be improved on. Finally, on patient/tumour demographics, more recent National Institute for Health and Care Excellence (NICE) guidance suggests that all patients with a diagnosis of anal cancer should be tested for HIV [34], [35] and this audit suggests further action is required to improve this measure. The number of patients undergoing a staging positron emission tomography/computed tomography (PET/CT) scan (39%) reflects the lack of validation of this modality in anal cancer. The recent NCCN guidelines have only just incorporated PET/CT into the workup of patients and even still only suggests ‘PET should be considered’ [36].

The audit also highlights the variability of management in early disease. The dose currently used in T1 and excised T2 tumours is currently heterogeneous. In T1 N0 tumours, 16 patients received 50.4 Gy in 28 fractions, and one patient each received the following doses: 36 Gy in 20 fractions, 40 Gy in 25 fractions, 30 Gy in 10 fractions and 52 Gy in 26 fractions in a split course treatment. Of the T2 N0 tumours post excision two patients received 50 Gy in 28 fractions, whereas one received 40 Gy in 28 fractions. This heterogeneity in dose makes outcomes in these groups difficult to assess. This is probably due to a lack of clear guidance or evidence in this setting. PLATO will address this issue with two of the studies under the PLATO umbrella investigating these patients. Regarding the issue of whether to treat nodes prophylactically in T1/T2 tumours, neither NCCN nor ESMO-ESSO-ESTRO guidance offers specific guidance due to a lack of sufficient evidence. The UK IMRT guidance suggests that in T1 tumours there may be patients where prophylactic nodal irradiation is not required. However, due to conflicting results in the literature regarding withholding prophylactic inguinal node irradiation in T2 tumours we suggest that T2 tumours do receive prophylactic nodal irradiation. Inguinal relapse rates in patients treated without prophylactic irradiation are reported in up to 22.5% [37], [38], [39], [40], [41].

UK IMRT guidance is in keeping with NCCN and ESMO-ESSO-ESTRO guidance, which suggests that mitomycin/5-fluorouracil or mitomycin/capecitabine should be used concurrently with radiotherapy [36], [42]. ESMO guidance notes that cisplatin, single agent or triplet combination, should not be used without good reason [43]. Ninety-three per cent of patients were treated according to that guidance. The few delivering single agent have noted that this was due to co-morbidities. Those using cisplatin have not documented reasons and it may be appropriate to investigate the rationale behind this.

Elderly patients should be offered radical treatment according to ESMO-ESSO-ESTRO with a recommendation that a reduction in treatment intensity should not be based on age alone but on a combination of co-morbidity and performance status [42]. There were 89% and 78% of patients over ≥75 years, who received full dose radiotherapy and CRT, respectively, which is encouraging. Performance status was not specifically requested and as such we cannot offer a reason why seven patients had reduced intensity treatment. Charnley *et al.* [44] published a small series using a reduced regimen of CRT for elderly and frail patients and showed excellent outcomes. However, in our audit a variety of different reduced dose CRT regimens was used. Further investigation of reduced intensity CRT in elderly patients with poor performance status or co-morbidities could inform guidance in this group.

NRIG recommendations would suggest that all patients who would benefit from IMRT should receive treatment with this technique. Due to the results of the RTOG 0529 study, node-positive patients will probably have reduced toxicity with IMRT and as such IMRT should be implemented, delivered and audited, to assess toxicity, in this group. The limitations to IMRT in the survey noted: capacity, expertise and time; as limiting factors to implementation. With the UK guidelines, additional education and support available with UK wide implementation we can address expertise. However, time and capacity are local issues that will need solutions to achieve the NRIG recommendation. It is encouraging that 87% of those treated with IMRT used the UK IMRT guidance. We hope publication of audit results will encourage the remaining few centres who practise outside the guidance to ensure homogeneous delivery and therefore assessment of outcomes using IMRT.

Our finding in this treated population-based audit is that acute toxicity is reduced in comparison with that reported in the ACT2 trial. This finding is similar to the findings of the RTOG 0529 trial, where acute toxicity was reduced in comparison with RTOG 9811 when delivered to 52 patients from 38 centres. It is interesting that the skin toxicity seems to be reduced through the use of IMRT (40% grade 3/4 in the two-phase conformal CRT group as compared with 25% in the IMRT treated patients, although not reaching statistical significance with such relatively small numbers, *x*^2^ *P* = 0.846), which is a similar finding to the RTOG 0529 study. The RTOG study chose not to power the trial on reduction in dermatological toxicity as they felt that the skin around the anal verge will probably continue to receive a similar dose with both techniques and as such a similar toxicity grading. However, the dose to the inguinal creases, which was previously a major source of morbidity, will be significantly reduced. This differentiation between toxicity at the two sites cannot be identified from this audit or the RTOG trial as all dermatological toxicity was graded together, but we could hypothesis this is the cause of the reduction in acute dermatitis.

Relative rates of diarrhoea and haematological grade 3/4 toxicity seemed to differ between the two-phase conformal CRT and IMRT treated cohorts, although none of these reached statistical significance, noting the relatively small numbers in each group. The more objective measure of frequency of treatment interruptions was also reduced, although again acknowledging that these are non-randomised data and differences in patient demographics (e.g. greater number of M1 patients in the two-phase conformal CRT group) may also contribute to differences in treatment tolerability and decision making. The main differences between the UK IMRT guidance and two-phase conformal [AQ1] are that the UK IMRT guidance allowed the use of capecitabine and the radiotherapy delivery technique. The increased grade 3 diarrhoea may be as a result of the capecitabine or the different radiotherapy technique. The increased thrombocytopenia with IMRT is more likely due to the dose-bathing that can occur due to the technique, as capecitabine is not associated with increased thrombocytopenia when used with pelvic radiotherapy [45]. The introduction of bone marrow constraints in the UK guidance could be considered. Another factor that must be considered when interpreting results is that the measures to support patients through radiotherapy have improved since ACT2 was undertaken; the significant improvement between toxicity in the published ACT2 data and the two-phase conformal [AQ1]in our audit is probably a reflection of this. The high volume centres in the audit were also more likely to deliver treatment with IMRT and as such the set up for support to minimise side-effects may have had some effect on the reduced toxicity. Further analysis of the data and a safety phase in ACT5 of PLATO study are planned to investigate the toxicity further.

There are limitations to this prospective audit. The annual incidence of anal cancer in the UK is about 1000 patients [46], as such we have captured over a 6 month period about half of all presenting patients. Although response rates to the audit, per centre, were 71%, which is comparable with other national RCR audits, the submission of 50% of all cases treated over the audit period is less than previous audits [47], [48]. The authors hypothesise that as the audit was entitled ‘The implementation of IMRT in anal cancer’, centres probably submitted more IMRT cases than conformal or palliative cases. As such, although the data may not represent overall anal cancer treatment, the IMRT findings are probably robust. Due to the multidisciplinary input of data and multiple patients from each centre there may be minor errors in data input. There was an absence of classifications for pathology and anatomy given to centres. Therefore the data received are the clinicians' understanding of the pathology and anatomy. In addition, due to a limited ability to clarify data submitted, some assumptions were made, which are all described above. However, in view of the rarity of anal cancer and the lack of data regarding capecitabine versus 5-fluorouracil beyond phase II studies, this dataset offers the ability to make some important observations in this disease during the implementation of IMRT and the set-up of subsequent clinical studies.

Future work will include further assessment of the acute toxicity data as the richness of information may contribute relevant information to further guidance and PLATO. In addition we plan to collect clinical response rates at 3 months and disease-free survival at 1 year to ensure clinical outcomes are maintained and repeat the EORTC questionnaire at 1 year, in order to obtain comprehensive data on long-term toxicity and patient-reported outcomes in a routine anal cancer population.

## Figures and Tables

**Table 1:** Patient demographics

	**ACT2 trial**∗	**All UK audit patients**	**Two-phase conformal CRT in UK audit**‡	**IMRT as per guidance in UK audit**‡
	(*n* = 246†)	(*n* = 242)	(*n* = 52)	(*n* = 157)
Age (years), median (range)	60	62 (29–90)	59 (38–87)	62 (29–88)
Age <65 years	75%	140 (58%)	30 (58%)	93 (59%)
Age ≥ 65 years	25%	102 (42%)	22 (42%)	64 (41%)
Gender				
Male	38%	64 (26%)	11 (21%)	47 (30%)
Female	62%	178 (74%)	41 (79%)	110 (70%)
Pre-treatment colostomy				
Yes	13%	38 (16%)	11 (21%)	24 (15%)
No	87%	204 (84%)	41 (79%)	133 (85%)
Pre-treatment PET/CT				
Yes	N/R	95 (39%)	19 (37%)	65 (41%)
No	N/R	147 (61%)	33 (63%)	92 (59%)
HIV status				
Positive	N/R	8 (3%)	1 (2%)	7 (4%)
Negative	N/R	84 (35%)	11 (21%)	67 (43%)
Not performed	N/R	150 (62%)	40 (77%)	83 (53%)
Smoking status				
Current smoker	N/R	58 (24%)	21 (40%)	38 (24%)
Ex-smoker (>6 months)	N/R	46 (19%)	9 (17%)	30 (19%)
Never smoked	N/R	95 (39%)	16 (31%)	60 (38%)
Do not know	N/R	43 (18%)	6 (12%)	29 (18%)

CRT, chemoradiotherapy; IMRT, intensity-modulated radiotherapy; PET/CT, positron emission tomography/computed tomography.

∗ Data from the arm receiving mitomycin concurrently and no maintenance chemotherapy.

† Patients in the mitomycin, no maintenance were used for demographic comparison.

‡ Excluding patients without inguinal irradiation.

**Table 2:** Tumour demographics

	**ACT2 trial**∗	**All UK audit patients**	**Two-phase conformal CRT in UK audit**‡	**IMRT as per guidance in UK audit**‡
	(*n* = 246†)	(*n* = 242)	(*n* = 52)	(*n* = 157)
Tumour type				
Squamous	95%	240 (99%)	52 (100%)	155 (99%)
Adenocarcinoma	0%	1 (0.004%)	0	1 (0.006%)
Small cell carcinoma	0%	1 (0.004%)	0	1 (0.006%)
Unknown	5%	0	0	0
Level of differentiation				
Well	12%	15 (6%)	2 (4%)	11 (7%)
Moderately	41%	96 (40%)	19 (37%)	63 (40%)
Poorly	30%	82 (34%)	18 (35%)	50 (32%)
Unknown	17%	49 (20%)	13 (25%)	33 (21%)
Site of primary tumour				
Canal	82%	184 (76%)	37 (71%)	122 (78%)
Verge	15%	25 (10%)	7 (14%)	14 (9%)
Distal Rectum	0%	20 (8%)	7 (14%)	11 (7%)
Peri-anal skin	0%	9 (4%)	1 (2%)	7 (4%)
No primary identified	0%	2 (1%)	0	2 (1%)
Other	2%	2 (1%)	0	1 (1%)
T stage				
T1	10%	28 (12%)	5 (10%)	15 (10%)
T2	40%	99 (41%)	22 (42%)	66 (42%)
T3	33%	61 (25%)	16 (31%)	39 (25%)
T4	13%	52 (21%)	9 (17%)	35 (22%)
Tx	4%	2 (1%)	0	2 (1%)
N stage				
Negative	63%	120 (50%)	24 (46%)	74 (47%)
Positive	31%	122 (50%)	28 (54%)	83 (53%)
Nx	5%	0	0	0
M stage				
M0	100%	221 (91%)	43 (83%)	148 (94%)
M1	0%	9 (4%)	4 (8%)	3 (2%)
Mx	N/R	12 (5%)	5 (10%)	6 (4%)

CRT, chemoradiotherapy; IMRT, intensity-modulated radiotherapy.

∗ Data from the arm receiving mitomycin concurrently and no maintenance chemotherapy.

† Patients in the mitomycin, no maintenance were used for demographic comparison.

‡ Excluding patients without inguinal irradiation.

**Table 3:** Chemotherapy regimens

	UK audit
	(*n* = 242)
Chemotherapy regimen	
MMC 5-FU	156 (64.5%)
MMC capecitabine	68 (28.1%)
Cisplatin/5-FU	3 (1.2%)
MMC alone	1 (0.4%)
5-FU alone	1 (0.4%)
Capecitabine alone	2 (0.8%)
Cisplatin/Etoposide	2 (0.8%)
No chemotherapy	8 (3.3%)
Cisplatin alone	1 (0.4%)

MMC, mitomycin; 5-FU, 5-fluorouracil.

**Table 4:** Comparison of grade 3+4 acute toxicity during chemoradiotherapy (CRT) seen in the ACT2 publication, all UK audit patients, UK audit patients undergoing ACT2 regimen and UK audit patient treated in keeping with UK intensity-modulated radiotherapy (IMRT) guidance

	**ACT2 trial**∗	**All UK audit patients**	**Two-phase conformal CRT in UK audit**	**IMRT as per guidance in UK audit**
		(*n* = 199 non-haematological;		(*n* = 127 non-haematological;
	(*n* = 472†)	*n* = 192 haematological∗‡)	(*n* = 45∗‡)	*n* = 120 haematological∗‡)
Non-haematological§	294 (62%)	87 (44%)	22 (49%)	51 (40%)
Gastrointestinal	75 (16%)	26 (13%)	5 (11%)	17 (13%)
Nausea	10 (2%)	6 (3%)	2 (4%)	4 (3%)
Vomiting	9 (2%)	4 (2%)	1 (2%)	3 (2%)
Diarrhoea	44 (9%)	18 (9%)	2 (4%)	13 (10%)
Stomatitis	14 (3%)	5 (3%)	1 (2%)	3 (2%)
Other gastrointestinal	16 (3%)	1 (1%)	0	1 (1%)
Skin	228 (48%)	60 (30%)	18 (40%)	32 (25%)
Pain	122 (26%)	28 (14%)	6 (13%)	16 (13%)
Cardiac	7 (1%)	3 (1%)	0	3 (2%)
Other non-haematological	34 (7%)	8 (4%)	2 (4%)	6 (5%)
Haematological§	124 (26%)	31 (16%)	6 (13%)	21 (18%)
Neutrophils	112 (24%)	25 (13%)	5 (11%)	15 (13%)
Platelets	21 (4%)	13 (7%)	3 (7%)	9 (8%)
Haemoglobin	2 (<1%)	2 (1%)	0	2 (2%)
Febrile Neutropenia	15 (3%)	2 (1%)	0	1 (1%)
Any toxic effect§	334 (71%)	104 (52%)	25 (54%)	62 (48%)

∗ Only the highest grade is counted and patients with more than one toxic effect of a particular grade were counted only once.

† Patients in the mitomycin/5-fluorouracil arm only were used for toxicity comparison.

‡ Numbers and percentages based on patients with submitted toxicity.

§ Patients with more than one toxic effect counted only once.

**Table 5:** Comparison of radiotherapy interruptions, chemotherapy completion and treatment deaths seen in the ACT2 publication, all UK audit patients, UK audit patients undergoing ACT2 regimen and UK audit patients treated in keeping with UK intensity-modulated radiotherapy (IMRT) guidance

Percentage (with 95% confidence interval)	ACT2 trial	All UK audit patients	Two-phase conformal CRT in UK audit	IMRT as per guidance in UK audit
*n*	940∗	242	52	157
Radiotherapy courses with interruptions	15%	N/A†	4 (8%)	7 (4%)
Patients completing planned radiotherapy dose (irrelevant of gaps)	N/R	227 (94%)	47 (90%)	150 (96%)
Patients completing planned chemotherapy	77%	201 (83%)	45 (87%)	131 (83%)
Treatment deaths	<1%	2 (1%)	2 (4%)	0

∗ Data from all patients within ACT2 were used for comparison as data for specific groups were not available.

† As includes palliative and lower dose patients not calculated.
