# Supplementary material for: Initial Results from the Royal College of Radiologists' UK National Audit of Anal Cancer Radiotherapy 2015
Source: Clin Oncol (R Coll Radiol). 2017 Mar;29(3):188–97. doi: 10.1016/j.clon.2016.10.005 (PMC5304408; doi:10.1016/j.clon.2016.10.005)
Supplement: Supplementary file 1 [file mmc1.pdf]

Supplementary Material

Survey Results 2013

38 of 56 centres responded.

1) How does your centre currently deliver anal cancer chemoradiation?

- 37% delivering IMRT on all or selected patients.
- 63% delivering ACT2 style treatment entirely.

2) Those delivering IMRT were asked what dose they use (free text):

- 52 in 28
- 50.4–55 depending on T stage
- RTOG 0529 doses
- As per ACT II
- 50.4 to tumour, 39.2 in 28 to nodes
- As per UK guidelines

3) Option for documenting reasons for having not implemented IMRT (free text):

- Experience, staff and machine capacity for development
- Medical physics support
- Capacity
- We are just starting to use IMRT but at the moment lack both capacity and expertise

4) Would your centre be prepared to implement IMRT using guidelines developed by the colorectal CSG?

- 100% were happy to implement using national guidance.

5) How long would it take for your centre to treat the first patient with the new technique?

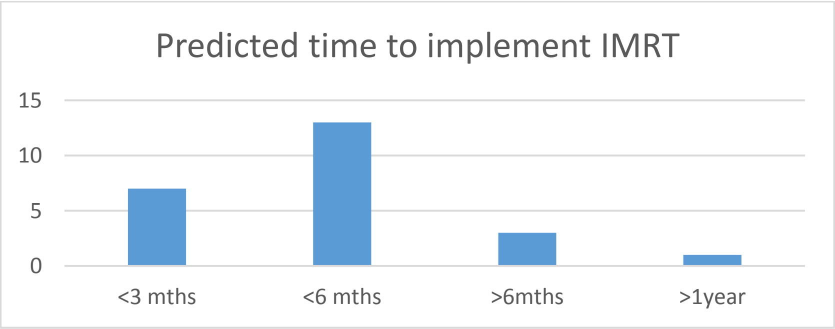

6) Would your centre be prepared to prospectively collect data at two timepoints (end of chemo/XRT and 3 months post XRT/chemo) in a minimum of five consecutive patients within a UK wide audit?

- 100% keen to participate in an UK audit of anal cancer.

7) Is PET/CT used in the workup for your anal cancer patients?

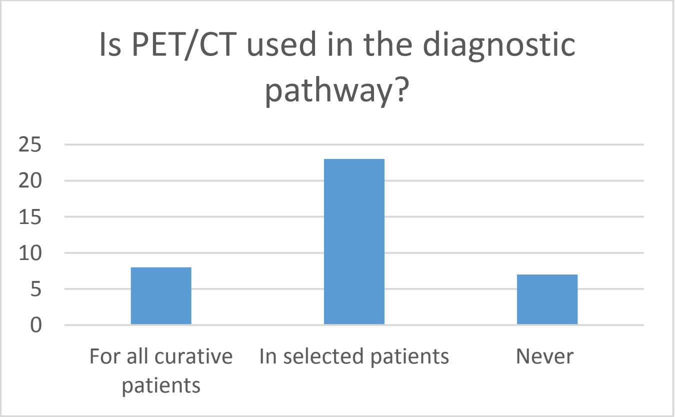

8) What criteria are used to select patients for PET/CT in your centre (free text)?

- Extensive disease
- Locally very advanced and/or extensive local adenopathy
- If areas of abnormality on staging CT requiring further assessment, that may affect treatment decisions
- Suspicious disease elsewhere
- All PTs other than very early
- If there is any doubt about extent of disease
- High risk t4, any T N2
- PET is available, but only done if there is equivocation on routine staging investigations.
- Larger cT2 +
- All advanced and those being considered for local RT
- T2 and above
- If they have equivocal paraaortic nodes i.e. ?distant mets
- For LN positive patients
- Risk of nodal disease
- If doubt about nodal status at MDT
- Currently all radical patients as part of an assessment
- Locally advanced disease
- Via the MDT if clinical useful
- At MDT, any suspicious lesions
- T3 and T4 patients
- To exclude suspicious mets seen on staging CT
- T3-4N+
- Based on staging CT and MR. PET in patients with suspected nodal or metastatic disease

9) How many patients with early (T1/T2 N0) and late (T3/T4 N+) see a year?

- T1/T2 [median (range)] = 7.5 (3–35)
- T3/T4 N+[median (range)] = 10 (3–30)

10) Would your centre be supportive of a study of dose de-escalation in early anal cancer?

- Yes – 31,
- No – 6,
- No reply – 1.

11) Would your centre be supportive of a study of dose escalation in locally advanced anal cancer?

- Yes - 36,
- No - 1
- No reply - 1.

12) Two investigative methods aiming to improve local control have been discussed, which of the below would be the preferred method at your centre?

- Dose escalation with additional fractions – 34
- Acceleration with 28 fractions delivered 6 days per week including Saturday – 2
- No reply – 2

13) Reasons?

- Staffing
- Do not work routinely on Saturdays
- Easier to facilitate
- Easier to facilitate
- Workforce issues
- Saturday treatment difficult practically and less acceptable on international stage
- We do not have provisions for Saturday treatment except for cord compression
- Unlikely that resources are available for weekend treatment
- Not currently routine to run a 6 day service although this may change so open to both
- Saturday treatments would present logistical problems.
- More rationale
- Saturday treatment challenging!
- Would be more internationally relevant.
- Capacity issues for Saturday treatment
- Limited Saturday resources to treat
- Weekend treatment not currently available
- 6 day working is an extra cost without necessarily better outcomes in a 35 day course
- Already much skin toxicity
- Difficult to arrange working for every Saturday
- Resource implications. Presume dose escalation is SIB not additional fractions?
- Results are very good now.

- We do not have routine radiotherapy during weekends. Would require a business case
- Acute reactions can be severe, working practice
- Lack of capacity at weekends may be an issue
- Logistics of routine weekend treatment
- We do not routinely treat on Saturdays. The cost implications would be prohibitive
- logistics of Saturday working tricky
- Radiotherapy machines only till 1pm on Saturday only for emergency
- Staffing over weekends would be a concern
- Saturday treatment not easy to set up but could be done
- Would be difficult to regularly staff the machines with radiographers on a Saturday
- No routine Saturday radiotherapy working at present

14) Any additional comments

- Very keen for agreed protocol to improve standards of treatment nationally
- We are in process of developing IMRT for our anal cancers
- We have treated 55 pts with IMRT so far.
- At our centre, we would be very keen to IMRT for treatment of anal canal cancer, in addition to the sites where it is currently used. Unfortunately due to current pressures within the department, particularly related to physics support, it would be difficult to introduce treatment right away, but it would be desirable to do so in the near future. Involvement in a national trial would be an excellent way to introduce new practice, and we would be very keen to be involved.
- We would be very keen to participate
- We are integrating IMRT volumes into our work instructions currently and hope to transition to IMRT based delivery of treatment in the next 3 months
- Our results are excellent. In my view we need to lower the acute and late toxicity by changing the chemotherapy schedule.
- Happy to support IMRT for this site which we were thinking of starting soon!
- Very good results with current protocol with only one salvage APR needed in 5 years.
- Time taken for IMRT is not included in our job plans.

Section 1: Centre ID and Patient ID

1. **Centre ID (allocated by the RCR)**

   ____

2. **Audit ID (allocated locally - please retain link to unique patient identifier e.g. NHS number)**

   ____

3. **Date of Birth (dd/mm/yy)**

   ____

Section 2: Information from initial assessment

4. **What is the patient’s sex?**
   - ❑ *Male*
   - ❑ *Female*

5. **What is the pathology of the anal carcinoma?**
   - ❑ *Epidermoid*
   - ❑ *Basaloid (cloacogenic)*
   - ❑ *Other -*

   If answer is other, please ask “What is the pathology of this anal carcinoma?”

6. **What is the level of differentiation on pathology?**
   - ❑ *Well differentiated*
   - ❑ *Moderately differentiated*
   - ❑ *Poorly differentiated*

7. **Does this patient have a pre-treatment stoma or is a pre-treatment stoma planned?**
   - ❑ *Yes*
   - ❑ *No*

8. **What is this patients smoking status?**
   - ❑ *Current smoker*
   - ❑ *Ex-smoker (>6months)*
   - ❑ *Never smoked*

9. **What is the patients HIV status?**
   - ❑ *HIV positive*
   - ❑ *HIV negative*
   - ❑ *HIV test not performed*

10. PLEASE UPLOAD EORTC QUESTIONNAIRE

Section 3: At treatment planning

11 **Was a diagnostic PET/CT performed?**
   - ❑ *Yes*
   - ❑ *No*

12 **What is the pathological T stage? (AJCC Cancer Staging 7th Edition)**

- ❑ *T1 (< 2 cm)*
- ❑ *T2 (>2 cm but <5 cm)*
- ❑ *T3 (>5 cm)*
- ❑ *T4 (Involving adjacent organs, e.g. vagina urethra and bladder. Direct invasion of the rectal wall, perirectal skin, subcutaneous tissue, or the sphincter muscle(s) is not classified as T4.)*

13 **What size is the primary tumour in short axis diameter (cm)?**

14 **What is the overall clinical and radiological N stage at treatment planning? (AJCC Cancer Staging 7th Edition)**

- ❑ *N0 (no regional lymph node metastasis)*
- ❑ *N1 (metastases in perirectal lymph node(s))*
- ❑ *N2 (metastases in unilateral internal iliac and /or inguinal lymph node (s)*
- ❑ *N3 (metastases in perirectal and inguinal lymph nodes and/or bilateral internal iliac and/or inguinal lymph nodes.*

15 **What is the radiological M stage? (AJCC Cancer Staging 7th Edition)**

- ❑ *Mx (cannot be sure regarding metastatic state due to discrepancies on imaging)*
- ❑ *M0 (no distant metastases)*
- ❑ *M1 (distant metastases present)*

16 **Where is / was the primary tumour situated?**

- ❑ *Anal verge*
- ❑ *Anal canal*
- ❑ *Peri-anal skin*
- ❑ *Distal rectum*
- ❑ *Other*

If the answer is other please ask "Where is / was the primary tumour situated?"

17 **Please note the number of involved nodes in each area according to clinical and radiological staging at the time of treatment planning.**

Left inguinal
Right inguinal
Left external iliac
Right external iliac
Left internal iliac
Right internal iliac
Left common iliac
Right common iliac
Mesorectal

XRT WK - DATE	**1**	**2**	**3**	**4**	**5**	**6 (optional)**	**12 (6wk R/V)**
	Grade	Grade	Grade	Grade	Grade	Grade	
Hb							
WCC							

18 **PLEASE COMPLETE AND UPLOAD TOXICITY SHEET**

Ischiorectal fossa
Left obturator
Right obturator
Para-aortic
Other
If the answer is other please ask "Where were the positive nodes situated?"

Section 3: On completion of chemoradiotherapy

Platelets							
Fatigue							
Dermatitis							
Nausea							
Vomiting							
Diarrhoea							
Sign of infection							
Anal Pain							
Others:							

Toxicity grading

		Grade 1	Grade 2	Grade 3	Grade 4
Blood	Haemoglobin	<LLN–10.0 g/dl	<10.0–8.0 g/dl	<8.0 g/dl transfusion indicated	Life threatening consequences; urgent intervention indicated.
	Neutrophils	<LLN–1.5 × 10e9/l	<1.5–1.0 × 10e9/l	<1.0–0.5 × 10e9/l	<0.5 × 10e9/l
	Platelets	<LLN–75.0 × 10e9/l	<75.0–50.0 × 10e9/l	<50.0–25.0 × 10e9/l	<25.0 × 10e9/l
Febrile neutropenia				ANC <1000/mm^3 with a single temperature of >38.3°C or a sustained temperature of >38°C for more than 1 h	
Fatigue		Fatigue relieved by rest	Fatigue not relieved by rest; limiting instrumental ADL	Fatigue not relieved by rest, limiting self care ADL	
Skin		Follicular, faint or dull erythema/ epilation/dry desquamation/ decreased sweating	Tender or bright erythema, patchy moist desquamation/ moderate oedema	Confluent, moist desquamation other than skin folds, pitting oedema	Ulceration, haemorrhage, necrosis

Gastrointestinal	Nausea	Loss of appetite without alteration in eating habits	Oral intake decreased without significant weight loss, dehydration or malnutrition	Inadequate oral caloric or fluid intake; tube feeding, TPN, or hospitalisation indicated	–
	Vomiting	1–2 episodes (separated by 5 min) in 24 h	3–5 episodes (separated by 5 min) in 24 h	> 6 episodes (separated by 5 min) in 24 h; tube feeding TPN or hospitalisation indicated	Life-threatening consequences; urgent intervention indicated
	Diarrhoea	Increase of <4 stools per day over baseline; mild increase in ostomy output compared with baseline	Increase of 4–6 stools per day over baseline; moderate increase in ostomy output compared with baseline	Increase of 7 stools per day over baseline; incontinence; hospitalisation indicated; severe increase in ostomy output compared with baseline; limiting self care ADL	Life-threatening consequences; urgent intervention indicated
Anal pain		Mild pain	Moderate pain; limiting instrumental ADL	Severe pain; limiting self care ADL	

19 **What chemotherapy regimen was planned?**

- ❑ *MMC and 5-FU*
- ❑ *MMC and capecitabine*
- ❑ *MMC alone*
- ❑ *5-FU alone*
- ❑ *Capecitabine alone*
- ❑ *Cisplatin and 5-FU*
- ❑ *Cisplatin/MMC/5-FU*
- ❑ *Other*

If they answer other please ask "What regimen was planned?"

20 **Was chemotherapy completed as planned?**

- ❑ *Yes*
- ❑ *No*

If the answer is no please ask "Why was chemotherapy not delivered as planned?"

21 **What radiotherapy delivery technique was used?**

- ❑ *IMRT*
- ❑ *VMAT / IMAT*
- ❑ *Tomotherapy*
- ❑ *ACT II regimen*
- *Other*

If the answer is other please ask "What radiotherapy delivery technique was used?"

22 **Please give the start date of radiotherapy**

Calendar?

23 **Please give the last date of radiotherapy**

Calendar?

24 **What was the planned dose and dose per fraction to prophylactic nodes**

- ❑ *Dose (Gy)*
- ❑ *No of fractions*

25 **What was the planned dose and dose per fraction to involved nodes?**

- ❑ *Dose (Gy)*
- ❑ *No of fractions*

26 **What was the planned dose and dose per fraction to primary tumour?**

- ❑ *Dose (Gy)*
- ❑ *No of fractions*

27 **What was the planned dose delivered in its entirety (not taking into account gaps)?**

- ❑ *Yes*
- ❑ *No*

28 **How many unplanned gaps were there during treatment (how many missed days were there?)**

- ❑ *0*
- ❑ *1-3*
- ❑ *4-6*
- ❑ *>7*

If answered any number other than 0 please ask "What was the cause of the gaps?"
